# Engineered Phage Enables Efficient Control of Gene Expression upon Infection of the Host Cell

**DOI:** 10.3390/ijms26010250

**Published:** 2024-12-30

**Authors:** Ting Wei, Wangsheng Lai, Qian Chen, Chenjian Sun

**Affiliations:** 1 CAS Key Laboratory for Quantitative Engineering Biology, Shenzhen Institute of Synthetic Biology, Shenzhen Institutes of Advanced Technology, Chinese Academy of Sciences, Shenzhen 518055, China; ws.lai@siat.ac.cn (W.L.); qian.chen1@siat.ac.cn (Q.C.); cj.sun@siat.ac.cn (C.S.); 2 University of Chinese Academy of Sciences, Beijing 100049, China

**Keywords:** engineered phage, inducible gene expression, phage shock protein, sigma factor, directed evolution

## Abstract

Recently, we developed a spatial phage-assisted continuous evolution (SPACE) system. This system utilizes chemotaxis coupled with the growth of motile bacteria during their spatial range expansion in soft agar to provide fresh host cells for iterative phage infection and selection pressure for preserving evolved genes of interest carried by phage mutants. Controllable mutagenesis activated only in a subpopulation of the migrating cells is essential in this system to efficiently generate mutated progeny phages from which desired individuals are selected during the directed evolution process. But, the widely adopted small molecule-dependent inducible system could hardly fulfill this purpose because it always affects all cells homogeneously. In this study, we developed a phage infection-induced gene expression system using modified *Escherichia coli* (*E. coli*) phage shock protein operon or sigma factors from *Bacillus subtilis*. Results showed that this system enabled efficient control of gene expression upon phage infection with dynamic output ranges from small to large using combinations of different engineered phages and corresponding promoters. This system was incorporated into SPACE to function as a phage infection-induced mutagenesis module and successfully facilitated the evolution of T7 RNA polymerase, which generated diverse mutants with altered promoter recognition specificity. We expect that phage infection-induced gene expression system could be further extended to more applications involving partial induction in a portion of a population and targeted induction in specific strains among a mixed bacterial community, which provides an important complement to small molecule-dependent inducible systems.

## 1. Introduction

As natural vehicles for gene transport, phages have provided an excellent material to be engineered for various purposes [1]. Their genomes can be manipulated with rapidly developing molecular biological methods to carry tailor-designed genetic parts [2,3]. In previous studies, engineered phages have already been utilized in a number of research fields, including the development of various directed evolution methods [4,5,6,7]. Phage-assisted continuous evolution (PACE) is an outstanding paradigm that links the desired property of biomolecules to phage propagation via the activation of phage gene III (*gIII*) expression from an accessory plasmid to enable rapid rounds of evolution [4,5]. Combining the advantages of PACE and bacterial range expansion system [8,9], we developed another directed evolution method named SPACE, short for spatial PACE [7]. SPACE utilizes chemotaxis coupled with the growth of motile bacteria during their spatial range expansion in soft agar [8,9] to provide fresh host cells for iterative phage infection and selection pressure for preserving the evolved gene of interest carried by phage mutants [7]. It enables the visualization and separation of different evolutionary events and remarkably simplifies the operation steps by removing the requirements for special culturing or monitoring equipment [7]. In both PACE and SPACE, induced mutagenesis plays an essential role in producing mutated versions of the phage-borne target gene, which encode different variants of the biomolecule to be evolved for selection based on their activity to stimulate *gIII* expression. Ideally, the expression of mutator genes carried by the in vivo mutagenesis plasmid [10] in host cells needs to be activated only in bacterial cell subpopulations infected by phages and hence harboring the phage genome so as to efficiently generate mutated progeny phages. Unnecessarily activating the mutagenesis module in host cells before phage infection could lead to loss-of-function mutations in the genetic circuit on the accessory plasmid designed to select for desired property of biomolecules, or even vitally damage the host cells before phage infection occurs [10].

Inducible promoters generally provide a convenient option to achieve activation of gene expression with specific timing and strength. Small molecules, such as isopropylthio-β-galactoside (IPTG), arabinose, rhamnose, and anhydrotetracycline (aTc), often serve as inducers to specifically activate their corresponding promoters to start transcription of genes downstream [9,10,11,12]. However, because the small molecules freely diffuse to all cells in the culturing system, it is impossible to partially turn on the gene expression in only a portion of individual cells in a population. In PACE using an arabinose-inducible mutagenesis module, the influence of inevitable mutagenesis induced ahead of ideal timing in uninfected bacterial cells is partially compensated by constantly supplementing fresh cells from a separate set of continuous culturing device free of arabinose [4,5]. Yet in SPACE, due to its highly simplified setup [7], fresh host cell production and induced mutagenesis can hardly be spatially separated. Therefore, the mutagenesis module needs to be delicately designed so that the expression of mutator genes can be activated only in the desired subpopulation of the host cells.

Rather than small-molecule inducers affecting all cells homogeneously, engineered phages could be flexibly designed to deliver genetic parts into only targeted cells [1,13,14]. In terms of gene expression regulation, phage genomes can be modified to carry genes encoding different regulators. Such a design separates the genetic parts of regulators and promoters into different compartments before phage infection occurs, which effectively helps reduce undesired basal level expression if the regulator is an activator and possible toxicity against the host cell caused by some regulators [15]. Furthermore, the engineered phages can be designed as either reproducible or irreproducible [14], the former potentially serving as an autoinducer and the latter enabling partial regulation of only a portion of the whole population simply by adjusting the multiplicity of infection (MOI) to below 1. These advantages of engineered phages make them an important and promising medium for the regulation of gene expression aside from small molecule inducers.

In this study, we designed a phage infection-induced gene expression system with two classes, using the *Escherichia coli* (*E. coli*) phage shock protein (*psp*) operon and sigma factor and promoter pairs from *Bacillus subtilis*, respectively. The engineered phages in both classes enabled efficient control of gene expression upon infection of the host cell, and an example of developing a mutagenesis module with the *Bacillus* sigma factor in SPACE demonstrated improved performance in generating diverse mutations.

## 2. Results

### 2.1. Native psp Operon of E. coli Can Be Utilized to Develop Phage Infection-Induced Gene Expression

**Figure 1:**
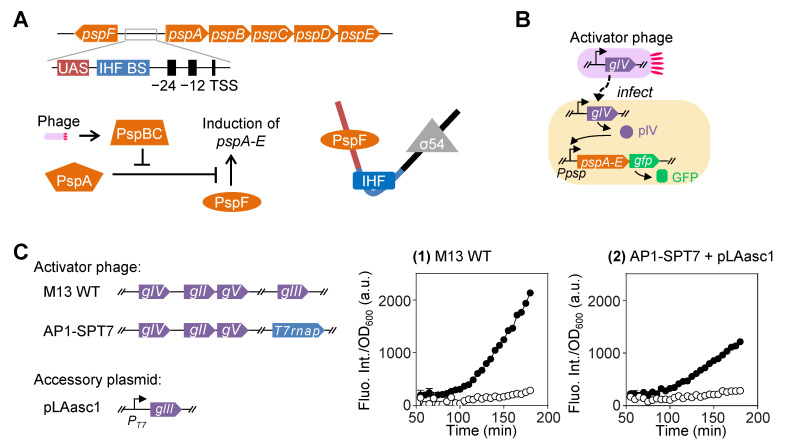
Application of the phage shock protein (*psp*) operon for construction of phage-inducible gene expression circuit. (**A**) Structure and working mechanism of *psp* operon in *E. coli*. Phage represents filamentous phage M13, of which the infection of *E. coli* cells activates the *psp* operon via a phage pIV-dependent signaling cascade. Psp, phage shock protein; UAS, upstream activating sequence; IHF BS, integration host factor binding site; TSS, transcription start site. (**B**) Design of a fluorescent gene expression system activated by phage infection using *psp* operon. The *E. coli* cells carry a reporter plasmid (RP), which harbors *gfp* under the control of the *psp* promoter. (**C**) Two versions of phages are used to activate the expression of *gfp* and the fluorescence intensity of their host cells across time after phage infection. Wild-type M13 was used in (**1**), and AP1-SPT7, which carries a T7 RNA polymerase gene in place of *gIII* and only produces infectious progeny phages in the presence of an accessory plasmid carrying *gIII* downstream of the T7 promoter, was used in (**2**). Native promoters are not annotated in the schematic diagram of the circuit design. The fluorescence intensity was normalized by the corresponding OD_600_ value at each time point. The starting time point of the plots was set at 50 min after phage inoculation when the OD_600_ values became significant enough to give stable normalized intensity values. Solid circle (●) represents the experimental group in which both *E. coli* FM15 cells and phages were added. Open circle (○) represents the control group in which only FM15 cells were added. The mean for two or three replicates is shown in the plot.

The *psp* operon is strongly induced by the filamentous phage secretin protein IV (pVI) as well as by a number of other stress conditions such as alkaline pH, ethanol, and defects in protein export [16,17,18,19]. The organization of *pspF* and *pspABCDE* (*pspA-E*) operon is shown in the upper part of Figure 1A. The *pspF* gene is located in the opposite direction of the other *psp* genes. The region between *pspF* and *pspA-E* contains a PspF-binding site named upstream activating sequence (UAS), an integration host factor (IHF) binding site, and a σ^54^-dependent promoter [20]. PspF serves as an activator that binds to UAS and interacts with the sigma factor with the help of IHF, which in turn promotes transcription [16,18,21,22,23,24] (Figure 1A). PspA acts as a negative regulator, while PspB and C work together as positive regulators by relieving the repression by PspA [21,25,26,27]. Upon the filamentous phage infection, the expression of *gIV* strongly activates the σ^54^-dependent promoter by a series of regulation steps [16,18,21] (Figure 1A).

We designed a reporter plasmid (RP) by placing a green fluorescent protein (GFP) gene downstream of *pspA-E* (Figure 1B, Table 1). Two versions of activator phages were used to induce the expression of the reporter gene via infection of the host cell: the wild-type (WT) M13 and a modified phage carrying the gene of T7 RNA polymerase (RNAP) in place of M13 *gIII* (Figure 1B,C). The latter version of activator phage was previously developed and used as a selection phage carrying a gene of interest, the T7 RNAP gene as a typical example, to be evolved in PACE and SPACE [4,7,28] (Figure 1C). Moreover, this modified M13 phage named AP1-SPT7 only produces infectious progeny phages in host cells harboring an accessory plasmid pLAasc1 to complement the expression of *gIII*, which is absent in the modified phage genome (Figure 1C).

The results of quantitative fluorescence tests in Figure 1(C1,2) showed that both versions of activator phages could effectively activate the expression of *gfp*, with 11.0- and 5.4-fold increase, for the WT and modified phages, respectively, in the normalized fluorescence intensity after induction by phage infection for 3 h. However, the background intensity values obtained from the control groups where no activator phages were inoculated signified considerable basal level expression in both groups of (1) and (2). That is, for longer than half of the whole duration of incubation, the basal level expression of the reporter gene maintained above 15% and 25% of the values under the induced condition for the two phage versions, respectively. We postulated that the relatively high basal level expression was caused by the long chain of interactions among different *psp* genes involved in the upregulation process.

### 2.2. Reduced psp Operon Effectively Lowers Basal Level Expression but Also Leads to Decreased Level of Desired Gene Expression upon Phage Infection

**Figure 2:**
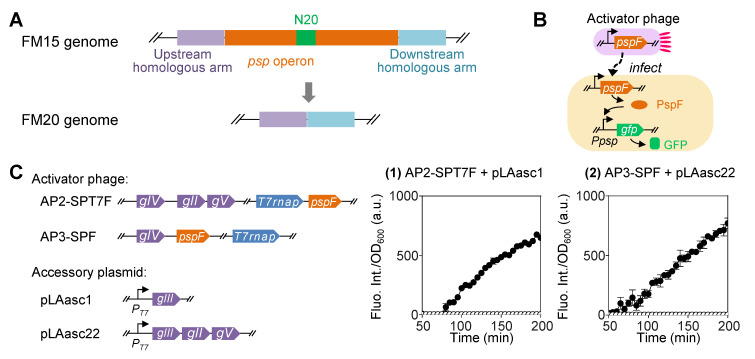
Modification of the *psp* operon for construction of phage-inducible gene expression circuit. (**A**) Construction of *E. coli* FM20 strain by deleting the native *psp* operon from FM15 genome with CRISPR-Cas system. FM20 was used as the bacterial host for the genetic circuit design using a modified *psp* operon. (**B**) Design of a fluorescent gene expression system activated by phage infection using the modified *psp* operon consisting only of the *psp* promoter without other *psp* genes and *pspF* carried by the activator phage. (**C**) Two versions of phages are used to activate the expression of *gfp* and the fluorescence intensity of their host cells across time after phage infection. AP2-SPT7F carries the T7 RNA polymerase gene followed by *pspF* in place of *gIII*. For further control of infectious progeny phage reproduction, *gII* and *gV*, two more phage genes were deleted, and *pspF* was inserted instead to construct another version of activator phage, AP3-SPF. Correspondingly, accessory plasmid pLAasc22 carrying *gIII*, *gII*, and *gV* downstream of the T7 promoter was constructed to enable the reproduction of AP3-SPF. Native promoters are not annotated in the schematic diagram of the circuit design. The fluorescence intensity was normalized by the corresponding OD_600_ value at each time point. Solid circle (●) represents the experimental group in which both *E. coli* FM15 cells and phages were added. Open circle (○) represents the control group in which only FM15 cells were added. The mean for three replicates is shown in the plot.

In order to lower the basal level expression, we sought to construct a reduced *psp* operon using solely PspF, rather than a PspBC-PspA-PspF cascade, to upregulate the transcription. We exploited the CRISPR-Cas9 system to delete the whole *psp* operon from the genome of the host bacterial strain *E. coli* FM15 [7] and derived a new host strain named FM20 (Figure 2A). In the meantime, we removed *pspA-E* from the reporter plasmid, leaving only the promoter region upstream of *gfp*. And *pspF* was placed in the genome of the activator phage (Figure 2B, Table 1).

Again, two versions of activator phages were derived from the modified M13 phage AP1-SPT7: AP2-SPT7F by inserting *pspF* immediately downstream of the RNAP gene, and AP3-SPF by replacing phage genes *gII* and *gV*, which are involved in the replication of the phage genome, with *pspF* (Figure 2C). The latter design was applied to test whether more stringent control over phage propagation could affect the process of phage-induced gene expression. Correspondingly, AP3-SPF relied on a new version of accessory plasmid pLAasc22, which carried *gIII*, *gII*, and *gV* to propagate in the host cells.

The results showed that the reduced *psp* operon relying solely on PspF for regulation indeed lowered the basal level expression. The fluorescence intensity of the control group was non-detectable throughout the test. Groups (1) and (2) exhibited 82.1- and 38.1-fold increases, respectively, in the normalized fluorescence intensity after induction by phage infection for 3 h (Figure 2C). Group (2) with more stringent control over activator phage propagation presented a closer to a linear tendency in the changes of expression level but gave a lower fold change over time. However, the absolute values of the detected fluorescence intensity in both groups were much lower than those observed in previous designs using the complete *psp* operon. This could pose difficulties in obtaining reliable values since inevitable fluctuations in the culturing conditions might cause non-negligible variations.

### 2.3. Heterologous Sigma Factors Provide Both High Phage Infection-Induced Gene Expression and Low Basal Level Expression

**Figure 3:**
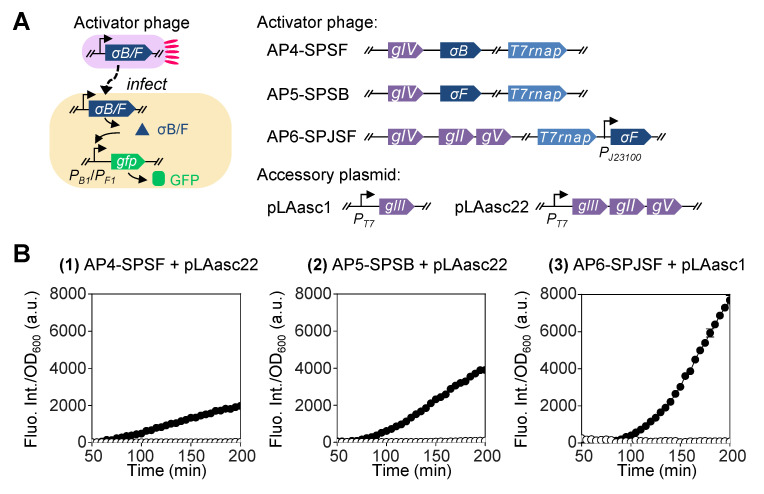
Construction of phage-inducible gene expression circuit using heterologous sigma factors. (**A**) Schematic design of a fluorescent gene expression system in *E. coli* activated by phage infection using heterologous sigma factors and promoters from *Bacillus*. These sigma factors are expected to bind the host core RNA polymerase (RNAP) and form a holoenzyme to recognize/transcribe specifically from its cognate promoter to yield an orthogonal expression system. (**B**) Three versions of phages are used to activate the expression of *gfp* and the fluorescence intensity of their host cells across time after phage infection. AP4-SPSF and AP5-SPSB carry the T7 RNAP gene in place of phage *gIII* and genes of *Bacillus* sigma factors B and F, respectively, in place of phage *gII*-*gV*. These two phages both rely on accessory plasmid pLAasc22 carrying *gIII*, *gII*, and *gV* downstream of the T7 promoter to reproduce. AP6-SPJSF was derived from AP1-SPT7 by inserting the gene of sigma factor F controlled by a strong synthetic promoter J23100 (https://parts.igem.org/Part:BBa_J23100 (accessed on 26 November 2020)) downstream of the T7 RNAP gene. This activator can also use accessory plasmid pLAasc1 to reproduce. Native promoters are not annotated in the schematic diagram of the circuit design. The fluorescence intensity was normalized by the corresponding OD_600_ value at each time point. Solid circle (●) represents the experimental group in which both *E. coli* FM15 cells and phages were added. Open circle (○) represents the control group in which only FM15 cells were added. The mean for three replicates is shown in the plot.

Next, we sought to improve the efficiency of the induced expression by introducing new parts into the system. Previous work reported the development of a toolbox using heterologous sigma factors and promoters from *B. subtilis* for orthogonal gene expression in *E. coli* [15]. We exploited two sigma factor and promoter pairs, σ_B_-*P_B1_* and σ_F_-*P_F1_*, reported with high specificity and transcriptional activity, to construct another set of activator phages and their corresponding reporter plasmids (Figure 3A).

The results confirmed that the orthogonality of the heterologous sigma factors and their promoters in *E. coli* cells led to almost negligible basal level expression. For groups (1) and (2) with more stringent control over phage propagation, the background fluorescence intensity was non-detectable for nearly 3 h (Figure 3B). During this process, the two groups exhibited 58.7- and 64.6-fold increases, respectively, in the normalized fluorescence intensity after induction by phage infection. For group (3), on the other hand, this fold change was elevated to as high as 444.8, with a relatively higher basal level expression observed in control during the first half of the incubation period but later dropped to no greater than 3% of those obtained under the induced condition.

### 2.4. SPACE Experiment Using a Mutagenesis Module Constructed with Heterologous Sigma Factor Produces Diverse Mutant Genotypes

Given that the heterologous sigma factors and promoters, especially σ_F_-*P_F1_,* could provide strong and specific transcriptional activation, we sought to utilize this system to improve the mutagenesis module for SPACE. In SPACE, initial phages carrying a wild-type target gene to be evolved infect host cells, and the expression of mutator genes induced by the infection leads to mutations in the target gene during the replication of the phage genome, producing variants of the target gene product. Desired variants with improved ability to activate the expression of *gIII* to produce infectious progeny phages, which in turn infect neighboring susceptible bacterial cells and repeat the process to finally form a fan-shaped infection zone in the bacterial lawn over the range expansion process of the host cells from the center to the entire of a soft agar plate [7]. Variants that do not lead to sufficient production of pIII and infectious progeny result in the formation of a typically much smaller fan-shaped pattern or no infection zone at all [7]. SPACE relies on the expression of the mutator genes to elevate the mutation rate during DNA replication so that the gene of interest carried by the initial phage genome could gain diverse mutations, from which those favoring progeny phage propagation could be selected and outcompete others. An ideal mutagenesis module for SPACE ought to present a negligible basal level expression of the mutator genes before phage infection occurs. While upon phage infection, the expression of the mutator genes is expected to be rapidly and strongly activated.

**Figure 4:**
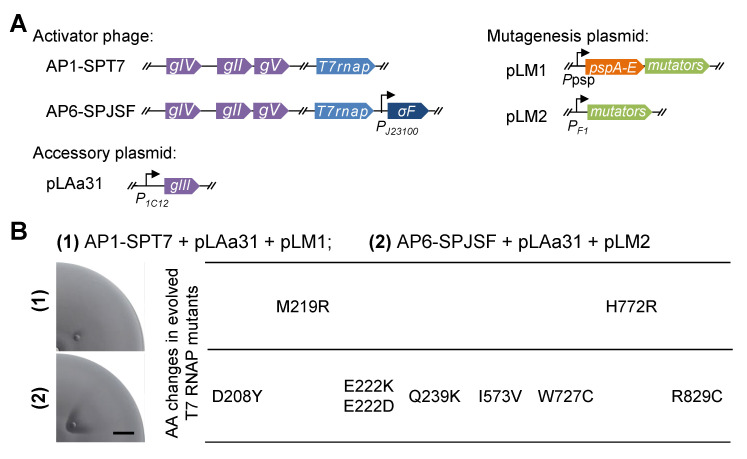
Spatial continuous directed evolution (SPACE) experiment using different versions of mutagenesis modules. (**A**) Two versions of activator phages carrying the T7 RNA polymerase (RNAP) gene as the target gene to be evolved and either the native phage *gIV* or *Bacillus* sigma factor gene of which the protein product can activate the expression of mutator genes from corresponding mutagenesis plasmids (pLM1/pLM2). (**B**) Upon phage infection, the mutagenesis process becomes active and leads to the production of various T7 RNAP mutants. Mutants with improved activity to recognize and transcribe from the target promoter 1C12 will lead to stronger infectious progeny phage propagation and will, in turn, produce a larger fan-shaped infection area with lower cell density on the bacterial lawn in the SPACE experiment. Evolved T7 RNAP mutant genes carried by sampled phages were sequenced, and the amino acid (AA) changes detected in these mutants are listed alongside the images of the SPACE agar plates from which the mutants were isolated. Photographs of a quarter of the semi-solid agar plate are shown in the Figure. Scale bar represents 1 cm.

Two versions of mutagenesis plasmids, using the native *psp* operon and σ_F_-*P_F1_*, respectively, were compared in a SPACE experiment evolving T7 RNAP to recognize a synthetic promoter 1C12 (Figure 4A). As it was proven in our previous work that a larger area of the phage infection zone produced in the bacterial lawn in SPACE signified the greater success of the evolution [7], pLM2 using σ_F_-*P_F1_* in group (2) clearly provided a more effective mutagenesis module for the system (Figure 4B). The results of sequencing the isolated mutants also showed that in group (2), more diverse mutations were generated, and they persisted throughout the evolution process, potentially providing more information to investigate the correlation between the genotype and the activity of T7 RNAP (Figure 4B).

## 3. Discussion

In this study, we designed and tested multiple versions of the phage infection-induced gene expression system. As a natural choice, the inherent *psp* operon in *E. coli* was first considered due to the readiness of required genetic parts in the host cells and, hence, the minimal requirements for modification [16]. This design based on native *psp* operon exhibited an acceptable range of expression level change when using the WT M13 phage, of which the reproducing capacity was fully exerted. While for the engineered activator phage with needs for complementary expression of pIII from accessory plasmid to complete packaging of infectious progeny phages [29,30], only half of the fold change in gene expression for WT was reached, making it impractical to be applied in genetic circuit designs. We postulated that it was caused by the high level of basal expression resulting from the complex interplay between different regulator proteins in the native *psp* system [31]. The follow-up modifications, including the construction of a *psp*-deleted *E. coli* strain, placing *pspF* into phage genome, and removing *pspA-E* from RP together, established a reduced *psp* operon, which, as expected, effectively lowered the basal level expression and significantly increased the fold change of induced expression. Although the absolute values of detected fluorescence intensity also largely decreased, this design using a reduced version of *psp* operon showed potential to be further improved probably by the engineering of PspF and its interaction with IHF and σ^54^ [20,23,32].

Compared with *psp* systems originated from *E. coli*, heterologous sigma factors from Gram-positive *B. subtilis* and their promoters exhibited excellent orthogonality toward the *E. coli* host. This system was previously developed based on the multi-subunit structure of bacterial RNA polymerases consisting of a core enzyme (α_2_ββ’ω) associated with a sigma subunit (σ) [15]. Together with their cognate promoters, *B. subtilis* sigma factors were introduced in the *E. coli* host and then bound to the host core RNAP to form a holoenzyme able to recognize and transcribe specifically from their cognate promoters [15]. These sigma factors generally activate gene expression to high levels [33,34], and most of the assayed naturally occurring promoters specific for sigma factors from *B. subtilis* are not recognized by *E. coli* sigma factors [15]. Combining these features, the tested heterologous sigma factors provided both high phage infection-induced gene expression and low basal level expression. This type of regulation toolbox could be further explored to enable the construction of complex synthetic systems.

Our trial of using a newly constructed mutagenesis module in SPACE based on the heterologous sigma factor from *B. subtilis* successfully revealed mutation sites in T7 RNAP that had not been studied in other works or detected in our previous SPACE experiments [7], such as D208Y, Q239K, I573V, W727C, R829C, suggesting the potential of this new module with excellent orthogonality toward the host to facilitate more thorough exploration for important sites influencing the functions of this widely utilized protein. These mutations possibly contribute to the fitness of the evolved T7 RNAP mutants due to the function of the domains where they are located. D208 and Q239 are located in the N-terminal domain (residues 1-310), which was suggested to be responsible for binding single-strand RNA, and residues 232–242 form an ‘‘intercalating hairpin’’ responsible for opening the promoter (unwinding) [35]. I573 and W727 are located in the fingers subdomain (residues 541–737 and 771–778), which makes most contacts between the template base that pairs with the incoming NTP and with the template strand immediately downstream of the templating base. R829 is located in the palm subdomain (residues 386–448, 532–540, and 788–838), which makes contact with the upstream region of the template strand and is important for positioning the template for catalysis. Moreover, mutations Q239L/R and V574A were detected in a PACE experiment that aimed to evolve T7 RNAP to recognize the T3 promoter [4], suggesting these residues, and perhaps their neighboring ones, contributed to the altered specificity of promoter recognition. In previous studies, we used PACE and SPACE to evolve T7 RNAP to recognize synthetic promoters [7] and to readthrough terminator-like sequences in order to reduce byproducts caused by immature termination during in vitro transcription (IVT) [28]. We obtained a library of T7 RNAP mutants with modified properties that, according to varying demands, could be potentially applied in different fields, such as manufacturing therapeutic RNAs by IVT [36]. We expect that our directed evolution systems could be further upgraded taking advantage of the new phage infection-induced mutagenesis module to generate more versatile mutant libraries of important biomolecules like T7 RNAP for in-depth structural analyses and practical applications.

In this study, we used two main types of phages, the WT, and the engineered versions missing essential phage genes, as vectors to carry factors that are able to induce desired gene expression. We have demonstrated that by adjusting the stringency of the control over the reproduction of engineered phages, the profile of phage infection-induced target gene expression could be altered. If the accessory plasmids for complementary expression of necessary phage genes are not introduced into the host cells, engineered phages would be irreproducible [4]. Alternatively, to simplify the delivered genome and more thoroughly remove the reproducibility, phagemids could be used instead [14]. Such flexibility in using phages as a delivery vector offers more space for the manipulation of the genetic circuits according to researchers’ demands. Other than activating gene expression simultaneously and homogeneously in all cells by using small molecule-dependent systems, partial induction in a portion of a population and targeted induction in specific strains among a mixed bacterial community can also be realized using the phage infection-induced gene expression system. This will potentially enable applications such as simulating the differential gene expression in bacterial cells commonly observed during biofilm formation [37], spatial organization for interspecific metabolic cooperation and cellular differentiation [38,39,40], or regulating gut bacterial community [41]. Moreover, we expect that by combining the advantages of small molecule-dependent and phage infection-induced systems, more complex regulation networks of gene expression could be designed and constructed.

In conclusion, we developed an inducible gene expression system with dynamic output ranges from small to large using different versions of engineered phages and their corresponding promoters. This system enabled efficient control of gene expression upon phage infection and could be utilized in designs of a number of different synthetic systems, such as mutagenesis modules for directed evolution.

## 4. Materials and Methods

### 4.1. Bacterial Strains, Plasmids, and Media

*E. coli* strains were used for different purposes: DH5α (TransGen, Beijing, China) was used for molecular cloning and construction of plasmids; FM15 [7] was used for phage propagation of M13 and its modified variants and SPACE experiments. FM20 was constructed by editing the genome of FM15 and used for induction tests of the reduced *psp* operon. Wild-type M13 and selection phages with M13 backbone [28] were used as templates to construct different phage variants carrying different transcriptional factors for induction of gene expression. Plasmids carrying target genes under the control of different inducible expression systems are listed in Table 1. Cells were cultured in Luria–Bertani medium (LB: 10 g L^−1^ NaCl, 10 g L^−1^ tryptone, 5 g L^−1^ yeast extract). LB containing 0.25% (*w*/*v*) agar (Huankai, Guangzhou, Guangdong, China) was used for SPACE experiments. Antibiotics, including chloramphenicol (25 μg mL^−1^), tetracycline (15 μg mL^−1^), carbenicillin (50 μg mL^−1^) and spectinomycin (100 μg mL^−1^) were added where appropriate.

### 4.2. Genome Editing for Construction of FM20

FM20 was constructed from FM15 by deleting the *psp* operon in the genome of the latter. A CRISPR-Cas9 system [42,43] was designed to specifically cleave a site located in the middle of the *psp* operon and to delete the whole operon by homologous recombination with 646 bp and 717 bp, respectively, upstream and downstream of the entire operon. The CRISPR-Cas9 system consisted of two plasmids: pCassac carrying, in order, the origin of replication oriR101, chloramphenicol resistance gene, Spy Cas9 gene, recombination genes gam, bet, and an exonuclease gene under control of an inducible promoter *P_BAD_*, *sacB*, gRNA scaffold targeting pMB1 with an N20 sequence CTATCGTCTTGAGTCCAACC, *rhaSR*; and pTarget carrying, in order, origin of replication pMB1, *aadA*, gRNA scaffold targeting *psp* operon with an N20 sequence CTGCATTACAGCAATCGTTC, *gfp* following a *P_λ_*, *rrnB* terminator. One round of transformation was carried out to introduce pCassac into chemically competent FM15 cells. Desired transformants were picked up and cultured overnight in LB broth at 37 °C with 200 rpm of shaking. One microliter of this culture was inoculated into 3 mL LB and shaking incubated until its optical density at 600 nm (OD_600_) reached 0.3. The culture was 1:30 diluted with 50 mL LB L-arabinose (10 mM) and incubated till its OD_600_ reached 0.3 again. This culture was transferred into a Corning centrifuge tube and kept on ice for 30 min, and then centrifuged at 4 °C and 2500× *g* for 10 min. The supernatant was carefully discarded, and 20 mL of pre-cooled bacteria-free MiliQ water was added to resuspend the pellet. The resuspension and centrifugation cycles were repeated three times. Then, 1 mL of pre-cooled water was added to resuspend the resulting electrocompetent cells. Then 600 ng of pTarget, 600 ng of DNA amplicon containing a total of 1363 bp of the upstream and downstream homologous sequences in order (Supplementary Materials), and 100 μL of the electrocompetent cells were mixed and added to a pre-cooled 0.1 cm-gap sterile electroporation cuvette (Bio-Rad, Hercules, CA, USA). The electroporation was carried out at a voltage of 1.8 kV. After this step, 1 mL of LB was added into the cuvette to resuspend the cells and then transferred into a 1.5 mL centrifuge tube. Then, this suspension was incubated at 37 °C with 200 rpm of shaking for 1 h, and then 100 μL of the suspension was plated on LB agar. Recombinants were screened by polymerase chain reaction (PCR) to confirm the deletion of the *psp* operon. Clones with their *psp* operon successfully removed were inoculated into LB broth supplemented with chloramphenicol and 10 mM rhamnose to induce cleaving of pTarget. After overnight shaking incubation at 37 °C, cultures were plated on LB agar supplemented with chloramphenicol. Clones without expression of *gfp* carried by pTarget were picked up for the following step. The removal of pTarget was further confirmed when the picked clones failed to grow on LB agar supplemented with spectinomycin. These confirmed clones were further cultured in antibiotic-free LB broth supplemented with 0.5% (*w*/*v*) glucose overnight, and then 100 μL of 10^4^-fold diluted culture was plated on LB agar supplemented with 0.5% glucose and 1% sucrose. Colonies were picked up and cultured overnight, and it was further confirmed that pCassac was cured when they failed to grow on LB agar supplemented with chloramphenicol. A round PCR was carried out again to confirm that the *psp* operon had already been deleted from the genome of FM15, and this resulting strain was named FM20.

### 4.3. Molecular Cloning for Construction of Plasmids and Modified Phages

Reporter plasmids contained *gfp* under the control of *E. coli psp* promoter or exogenous promoters from *Bacillus*. These vectors were constructed with Gibson assembly (NEB, Ipswich, MA, USA) or ClonExpress II One Step Cloning (Vazyme, Nanjing, China) kits. PCRs were carried out with PrimeSTAR Max (Takara Bio, Kusatsu, Japan) following the manufacturers’ instructions. To construct activator phages, double-stranded M13 genomes (selection phages from [7]) were used as backbone instead of normal plasmids. As described previously [7], these modified phage genomes were inserted with the abovementioned activator genes and then transformed into host strain FM15 carrying an accessory plasmid that encoded necessary genes (*gIII*, *gII*, and *gV*) to facilitate the packaging of phage particles. Plasmids and phages used and constructed in this work are listed in Table 1.

### 4.4. Quantitative Fluorescence Tests

For measurements of fluorescence intensity, M9 supplemented with 0.1% (*w*/*v*) casamino acids (CAA) and 0.4% (*w*/*v*) glucose was used. Antibiotics were added where appropriate. FM15 or FM20 strains containing reporter and activator pairs were cultured in the M9 medium overnight at 37 °C and 100-fold diluted in fresh medium for continued cultivation until its OD_600_ reached 0.3~0.4. This culture was again 20-fold diluted and cultivated till its OD_600_ reached 0.2. Then 20 μL of the culture was transferred to each well of a 96-well microplate with clear flat bottom and black walls (Corning, Corning, NY, USA) containing 180 μL of M9 medium supplemented with appropriate antibiotics and 0.4 mM of isopropylthio-β-galactoside (IPTG) where induction of gene expression was necessary. The microplate containing bacterial cultures was loaded into a Synergy H1 multi-mode microplate reader (Biotek, Winooski, VT, USA) and continuously cultured at 37 °C with a high shaking speed. OD_600_ and fluorescence (Ex/Em = 485/515 nm, Gain 75) were measured every 5 min. The fluorescence intensity was normalized by the OD values, and samples without the addition of IPTG were used as a negative control.

When using activator phages to induce the expression of fluorescence, the overnight culture of bacteria was 100-fold diluted in fresh M9 medium, cultivated till its OD_600_ reached 0.3~0.4, and again cultured till its OD_600_ reached 0.2 after another 20-fold dilution. Then, 100 μL the bacterial culture was transferred to each well of a 96-well microplate and mixed with an equal volume of phage lysate with a titer of 10^9^ PFU mL^−1^ to get a multiplicity of infection (MOI) of approximately 10. Fluorescence intensity and OD_600_ were measured as described above.

### 4.5. SPACE

A SPACE experiment was performed to test the effect of using a *Bacillus* sigma factor and promoter pair for the activation of the mutagenesis step. This experiment was carried out following our previously reported protocol. Activator phages carrying the T7 RNA polymerase gene (AP1-SPT7 or AP6-SPJSF) and accessory plasmid (pLAa31) containing a T7 promoter variant 1C12 (TAATACGACCCACTTCAGGGAGA) were used for the evolution [7]. Compared to the T7 promoter, 1C12 has a one-base difference in the region recognized by the specificity loop of T7 RNAP and a two-base difference in the unwinding region. Soft LB agar was freshly prepared in 8.5 cm Petri dishes before each SPACE experiment. FM15 cells carrying both accessory plasmid (pLAa31) and mutagenesis plasmid (pLM1 or pLM2) were cultured until its OD_600_ ≈ 0.2, and 2 μL aliquots of the cell suspension were inoculated at the center of the agar plates. Two microliters of selection phages with a titer of approximately 5 × 10^8^ PFU mL^−1^ were inoculated 1 cm away from the center of the soft agar. The inoculated plates were incubated at 37 °C for 18–20 h, which was typically the duration of bacterial growth and phage propagation required for the formation of a clear fan-shaped infection zone.

After the incubation, plates were imaged using a Canon EOS 600D digital camera (Canon, Tokyo, Japan) with a Canon EFS 18–135 mm lens and an exposure setting of f11, 1/500s, ISO3200. The agar plates were illuminated by a white LED ring light with a diameter of 36 cm and 16 cm below [9]. For evolved phage sample collection, 5 μL of the soft agar containing bacteria and phages was aspirated with a pipettor from the end of each edge of the fan shape, added to 495 μL fresh LB broth, and mixed by vortex at low speed. This liquid sample was then filtered through a 0.22-μm pore size PES syringe filter to remove bacteria and stored at −20 °C before use.

For sequencing the phage mutants, the collected samples after evolution were subjected to double-layer plaque assay and phage clone purification with host cells carrying the accessory plasmid. Purified phage clones were used for Sanger sequencing of the T7 RNAP gene.

## Figures and Tables

**Table 1:** Accessory plasmids, mutagenesis plasmids, and engineered phages used in this study.

Name of Vector	Class	Antibiotic Resistance	Origin of Replication	Promoter	Genes	Source
*Plasmids*						
pLAa1	Accessory plasmid	Spe	pUC	*P_T7_*	*gIII*	[7]
pLAa31	Accessory plasmid	Spe	pUC	*1C12*	*gIII*	[7]
pLAasc1	Accessory plasmid	Carb	SC101	*P_T7_*	*gIII*	[7]
pLAasc2	Accessory plasmid	Carb	SC101	*1C12*	*gIII*	[7]
pLAasc22	Accessory plasmid	Carb	SC101	*P_T7_*	*gIII-gII-gV*	This study
pLAa188a	Accessory plasmid	Carb	SC101	*P_psp_*	*gIII*	[7]
pLAc1	Activator plasmid	Spe	p15A	*P_lac_*	*pspF*	This study
pLM1	Mutagenesis plasmid	Chl	CloDF13	*P_psp_*	*pspABCDE-dnaQ926-dam-seqA*	[7]
pLM2	Mutagenesis plasmid	Chl	CloDF13	*P_F1_*	*dnaQ926-dam-seqA*	This study
pLRp1	Reporter plasmid (RP)	Chl	CloDF13	*P_psp_*	*pspABCDE-gfp*	[7]
pLRp2	RP	Chl	CloDF13	*P_psp_*	*gfp*	This study
pLRp4	RP	Chl	CloDF13	*P_B1_*	*gfp*	This study
pLRp5	RP	Chl	CloDF13	*P_F1_*	*gfp*	This study
*Engineered phages*						
AP1-SPT7	Activator phage		f1	*P_gIII_*	*T7 RNAP WT*	[7]
AP2-SPT7F	Activator phage		f1	*P_gIII_*	*T7 RNAP WT-pspF*	This study
AP3-SPF	Activator phage		f1	*P_gIV_/P_gIII_*	*pspF/T7 RNAP WT*	This study
AP4-SPSF	Activator phage		f1	*P_gIV_/P_gIII_*	*sigF/T7 RNAP WT*	This study
AP5-SPSB	Activator phage		f1	*P_gIV_/P_gIII_*	*sigB/T7 RNAP WT*	This study
AP6-SPJSF	Activator phage		f1	*J23100/P_gIII_*	*sigF/T7 RNAP WT*	This study

## Data Availability

The data that support the findings of this study are available from the corresponding author upon reasonable request.

## Supplementary Materials

The following supporting information can be downloaded at https://www.mdpi.com/article/10.3390/ijms26010250/s1.
