# Supplementary material for: Engineered Phage Enables Efficient Control of Gene Expression upon Infection of the Host Cell
Source: Int J Mol Sci. 2024 Dec 30;26(1):250. doi: 10.3390/ijms26010250 (PMC11720261; doi:10.3390/ijms26010250)
Supplement: Supplementary file 1 [file ijms-26-00250-s001.zip › ijms-3387607-supplementary.pdf]

Engineered phage enables efficient control of gene expression upon infection of the host cell

Ting Wei[1,2,#,*], Wangsheng Lai[1,#], Qian Chen[1], Chenjian Sun[1]

[1]Key Laboratory of Quantitative Synthetic Biology, Shenzhen Institute of Synthetic Biology, Shenzhen Institutes of Advanced Technology, Chinese Academy of Sciences, Shenzhen 518055, China
[2]University of Chinese Academy of Sciences, Beijing 100049, China

[#]equal contribution
*To whom correspondence may be addressed. Email: ting.wei@siat.ac.cn

SUPPLEMENTARY MATERIALS AND METHODS

>Homologous arms used in genome editing for construction of FM20
A total of 1,363 bp of the upstream and downstream homologous sequences in order, with the upstream homologous sequence highlighted by red font

TTGCCATGGATCATTACGCCGATAAGCCGGATTTAATGACGATGGCGAAAAGCCTCGCGGGCGGGATGCCGCTTTCGGGCGTGGTCGGTA
ACGCGAATATTATGGACGCACCCGCGCCGGGCGGGCTTGGCGGCACCTACGCCGGTAACCCGCTGGCGGTGGCTGCCGCGCACGCGGT
GCTCAACATTATCGACAAAGAATCACTCTGCGAACGCGCGAATCAACTGGGCCAGCGTCTCAAAAACACGTTGATTGATGCCAAAGAAAG
CGTTCCGGCCATTGCTGCGGTACGCGGCCTGGGGTCGATGATTGCGGTAGAGTTTAACGATCCGCAAACGGGCGAGCCGTCAGCGGCGA
TTGCACAGAAAATCCAGCAACGCGCGCTGGCGCAGGGGCTGCTCCTGCTGACCTGTGGCGCATACGGCAACGTGATTCGCTTCCTGTATC
CGCTGACCATCCCGGATGCGCAATTCGATGCGGCAATGAAAATTTTGCAGGATGCGCTGAGCGATTAATAGCCCAACGCAATAATGTCTGA
TGCGCTGCGCTTATCAGGCCTGCAAACGACGTATTGATTATGTATGCCGAATAAAGCATTCACGCCGCATCCGGCAAGTTGTATTGCTCAAC
TTCGCTAAATCTGGTGAAGAATTCACCATGAGCGGCGCTTATGCGCCGTTTTTTTTTCTGTCACACCTTATTTACATCCCCATAGATTATTTGC
GTCAGCTCACAAATACGCTTTTTCCCTGGTAAAAAATGATTTCCTGCGTGACTAAAACCCTTGTGCTCAATTGACAGTTTATTTTCTGCGGA
GTAGTCTCTCGTTTCATGGGACCGCTACCACGGAAAGGCAACATGAAACAGAAAATTACGGATTACCTGGACGAAATCTACGGTGGAACA
TTTACCGCAACTCATTTACAGAAACTTGTAACGCGTCTTGAGAGTGCGAAACGATTAATTACACAGCGACGTAAAAAACACTGGGATGAAA
GTGATGTCGTGTTAATTACCTATGCCGATCAATTTCACAGCAATGATTTAAAACCATTACCCACATTTAATCAGTTTTACCATCAATGGCTGCA
AAGCATTTTTTCACATGTTCATTTGTTGCCGTTTTATCCATGGTCATCTGATGATGGCTTTTCGGTAATTGATTATCATCAGGTCGCCAGTGAA
GCGGGGGAGTGGCAGGATATTCAGCAACTCGGTGAATGCAGTCATTTAATGTTTGATTTTGTCTGCAACCATATGTCGGCAAAAAGTGAAT
GGTTTAAAAACTATTTACAACAGCATCCAGGTTTTGAAGATTTTTTTATTGCCGTTGACCCGCAAACCGATCTCAGCGCCGTCACTC
